# Evaluation of the re-bond strength of debonded metal and ceramic brackets following Er: YAG laser treatment

**DOI:** 10.1186/s12903-024-04504-2

**Published:** 2024-06-20

**Authors:** Xiaowan Zheng, Xiaofeng Huang

**Affiliations:** Department of Stomatology, Beijing Friendship Hospital, Capital Medical University, No. 95 Yong’an Road, Xicheng District, Beijing, 100050 China

## Abstract

**Background:**

Failure of orthodontic bracket bonds is a common occurrence during orthodontic treatment. This study investigated the impact of Er: YAG laser-based removal of adhesive from the bases of metal and ceramic brackets for re-bonding.

**Methods:**

A total of 168 extracted premolars were collected from patients. 84 metal brackets were used to be bonded on the buccal surface of the premolars in Groups 1, 2, 3 and 4, while 84 ceramic brackets were applied in Groups I, II, III and IV. Group 1/I represented the initial bonding group, with Group 2/II being the re-bonding group with new brackets, while Groups 3/III and 4/ IV received recycled brackets treated by Er: YAG laser or flaming respectively. Both the first and second de-bonding were performed in all samples using a universal testing machine to determine the shear bond strength (SBS). The adhesive remnant index (ARI) was evaluated using a stereo-microscope. The new and the treated bracket bases were evaluated using scanning electron microscopy (SEM). Differences in initial bonding and re-bonding ability were analyzed through one-way ANOVAs, and differences in ARI were assessed with the Kruskal–Wallis test.

**Results:**

Greater amounts of adhesive residue were observed on ceramic brackets treated by laser. The SBS values for recycled metal brackets in Group 3 (26.13 MPa) were comparable to Group 1 (23.62 MPa) whereas they differed significantly from Group 4 (12.54 MPa). No significant differences in these values were observed when comparing the 4 groups with ceramic brackets. ARI score in Group 4 (2–3 points) differed significantly from the three other groups (*P* < 0.05). For Group I, II, III and IV, similar ARI scores were observed (*P* > 0.05). SEM analysis didn’t show apparent damage of bracket bases consisting of either metal or ceramic material treated by Er: YAG laser.

**Conclusions:**

Er: YAG laser treatment was superior to flame treatment as a means of removing adhesive without damaging the brackets. SBS values and ARI scores following Er: YAG laser treatment were similar to those for new brackets, offering further support for Er: YAG laser treatment as a viable means of recycling debonded brackets.

**Supplementary Information:**

The online version contains supplementary material available at 10.1186/s12903-024-04504-2.

## Introduction

The successful fixed orthodontic approaches depend on achieving robust bonding between the utilized orthodontic brackets and the underlying enamel such that they remain stable when exposed to orthodontic and masticatory stresses [1]. However, a range of factors have the potential to compromise the strength of this bond and result in premature adhesive failure, such as substrate contamination [2], composite thickness [3], or curing light output [4]. During treatment, replacement or re-bracketing becomes necessary. Given these factors, it may be possible to reuse these brackets as a means of lowering the associated treatment costs. Prior to re-bonding, however, it is vital that residual adhesives should be carefully and thoroughly removed from the base of the bracket without causing any damage to the bracket itself or altering the bracket slot dimensions. The most common clinical approaches used for adhesive removal include grinding, sandblasting, and burning [5–7].

Flame removal is the most common adhesive removal strategy. However, it can lead to the discoloration of the brackets, damage to the lock cover, and it is incompatible with the use of self-locking brackets. While, grinding can be highly effective, but it can also alter the shape of the bracket base, thereby leading to a reduction in bond strength. Although sandblasting can similarly be effective, but it is too cumbersome and time-consuming for clinical operation. Laser technologies are increasingly being embraced in the field of oral therapy [8–13]. In addition to enhancing enamel surface adhesion, laser treatments can be used to aid in the removal of adhesive from debonded brackets, then the treated brackets can be reused in the same patient [11, 14–16]. In prior study, an Er, Cr: YSGG laser was used at a wavelength of 2780 nm to remove adhesives from the bases of metal brackets while maintaining bonding strength sufficient for further clinical use [17]. Er: YAG lasers, which operate at 2940 nm, share a wavelength similar to that of the Er, Cr: YSGG laser, can effectively generate the requisite photothermal effect following their absorbance by water molecules and hydroxyapatite. To minimize the harm resulting from elevated surface temperature, a water jet spray is applied for cooling [18, 19].

Although laser are increasingly used in dental therapy, there has been relatively little researches focused on the application of Er: YAG laser as an approach to treat debonded brackets. Bracket base re-bonding strength has previously been the sole analyzed readout after treatment [14], and little is known about the morphological changes that take place in the bracket base and the surface of the bonded tooth. Here, an Er: YAG laser was utilized to remove adhesive from the bases of both metal and ceramic brackets and the re-bonding strength of these brackets was compared to the initial bonding strength. Bracket base morphology and tooth surface after debonding was carefully observed, and the results were compared to those associated with flame-based adhesive removal to evaluate the advantages of using an Er: YAG laser for bracket recycling.

## Materials and methods

### Tooth selection and storage

This experiment mainly used adhesive strength as the primary analysis indicator, and adopted the hypothesis test of population means in two groups. Drawing from the results of comparable published literature [14], it was assumed that the minimum difference in adhesive strength mean between the four groups was 1.14, and the standard deviation was 0.9. We selected *α* = 0.0083 (bilateral test), confidence = 1-*β* = 0.8, four equal sample sizes, using the following formula to estimate the sample size using PASS 2021 software. $\text{n}1=\text{n}2=\text{n}3=\text{n}4=2{\left[\frac{({\text{t}}_{{\upalpha }}+{\text{t}}_{{\upbeta }})\text{s}}{{\updelta }}\right]}^{2}$ (In the formula: ${t}_{\alpha }$=2.39, ${t}_{\beta }$=1.28; *s* was the standard deviation; *δ* was the mean difference).

**Fig. 1:**
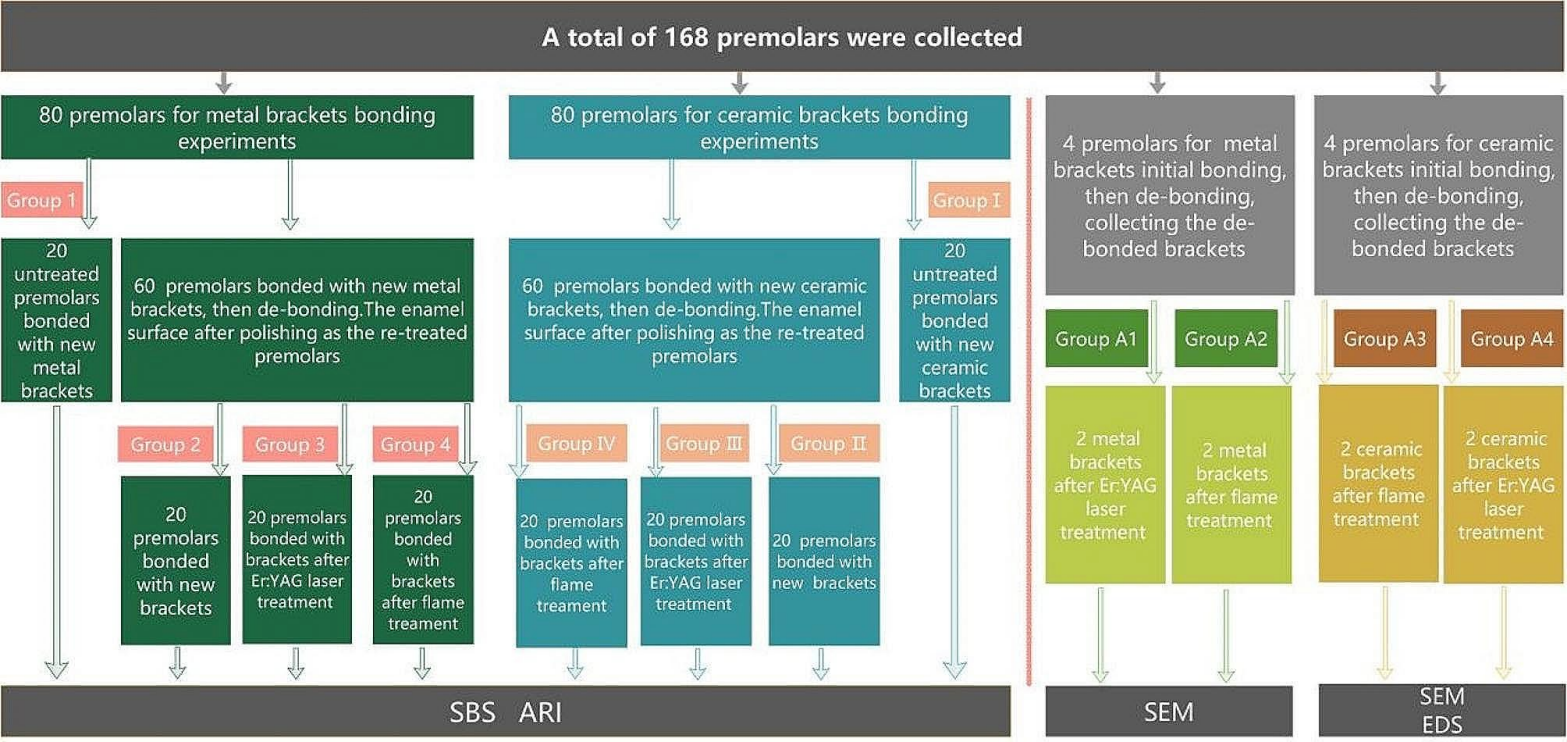
Experimental flowchart

Utilizing the Means menu in PASS 2021 under ‘Two-Sample T-Tests Allowing Equal Variance’ with these predefined parameters, it was determined that each group required a sample size of 17. Consequently, with eight groups each including 20 teeth, a total of 160 samples were enrolled in the study, ensuring both the accuracy and scientific rigor of the research findings. Scanning electron microscopy was used to observe the bracket bases after treatment with either flame or Er: YAG laser, thus an additional 8 teeth were included for debonded brackets, resulting in a total of 168 teeth. The protocol (No. 1835) was approved by the Bioethics Committee of Beijing Friendship Hospital Affiliated to Capital Medical University. The flowchart of the study is shown in Fig. 1.

168 teeth were selected and numbered, then grouped using random number method (using the random number formula in Excel software): 8 teeth were selected for scanning electron microscope (SEM) and energy dispersive x-ray spectroscopy (EDS) observation of bracket base. They were randomly divided into 4 groups, with 2 teeth in each group, namely Groups A1, A2, A3, and A4; the remaining 160 teeth were randomly divided into 8 groups, with 20 teeth in each group, were used to test bonding and re-bonding strength. The specific grouping situation is as follows:

Group A: 8 teeth for SEM observation. Group A1 (metal brackets treated by Er: YAG laser, *n* = 2), Group A2 (metal brackets treated by flame, *n* = 2), Group A3 (ceramic brackets treated by Er: YAG laser, *n* = 2), Group A4 (ceramic brackets treated by flame, *n* = 2),

Group 1: initial bonding (new metal brackets, *n* = 20).

Group 2: re-bonding (new metal brackets, *n* = 20).

Group 3: re-bonding (reused metal brackets by Er: YAG laser treatment, *n* = 20).

Group 4: re-bonding (reused metal brackets by flame treatment, *n* = 20).

Group I: initial bonding (new ceramic brackets, *n* = 20).

Group II: re-bonding (new ceramic brackets, *n* = 20).

Group III: re-bonding (reused ceramic brackets by Er: YAG laser treatment, *n* = 20).

Group IV: re-bonding (reused ceramic brackets by flame treatment, *n* = 20).

### Tooth preparation

#### Tooth preparation for initial bonding

All the soft tissue residue were removed from the premolars extracted for orthodontic purposes. After examined by a stereo-microscope at 10 magnification to ensure they had no obvious enamel cracks, caries, or restorations, the premolars were immersed for 24 h in distilled water at 37 °C, followed by transfer into a 1% thymol solution to prevent the growth of bacteria. Distilled water was refreshed once a week, and teeth were stored for no more than 6 months. Before bonding, the teeth were cleaned using pumice and fluoride-free toothpaste (Proxyt, Ivoclar, Schaan, Liechtenstein) for 20 s with a rubber cap (P-C505, TPC, Guanzhou, China), then rinsed with distilled water and dried with air. The teeth were etched for 60 s with 35% phosphoric acid gel (Gluma Etch, Kulzer, Wehrheim, Germany), followed by rinsing for 5 s with distilled water and drying for 15 s. Transbond XT primer and Transbond XT (3 M Unitek, Manrovia, California, USA) were used to bond 80 new metal brackets (Z2, Hangzhou Xingchen 3B Dental Instrument & Material Co. Ltd, Hangzhou, China) and 80 new ceramic brackets (polycrystalline alumina, Hangzhou Xingchen 3B Dental Instrument & Material Co. Ltd, Hangzhou, China) respectively.

20 teeth in Group 1 and 20 teeth in Group I were selected directly for initial shear bond strength (SBS) testing and the adhesive remnant index (ARI) scores recording.

#### Tooth preparation for re-bonding

The surfaces of another 120 initial bonded teeth for re-bonding were prepared as follows:

1. Debonding: Brackets were removed from these teeth with de-bonding pliers.
2. Tooth surface re-treating: Any adhesive remaining on the teeth was cleaned using an adhesive removal clamp and fluoride-free paste to polishing.

### Bracket base cleaning

After de-bonding, 44 metal brackets in Group A1, A2, 3, 4 and 44 ceramic brackets in Group A3, A4, III, IV were selected and separated equally into two groups:

1. Er: YAG laser (LiteTouch™, Sylleron, Yokneam, Israel) group (Group A1, A3, 3, III): For this treatment, samples were held in place using metal tweezers, and a laser at a wavelength of 2940 nm was used with the following settings: 300 mJ energy, 20 Hz frequency, 6 W output average power, 223 µs pulse duration, 6/8 water volume, and a scaler tip of 1.3 × 19 mm (serial: AS7071X), 22.61 J/cm^2^ energy density. The operator fixed the brackets onto the dental chair workbench, held the laser handle tightly against the dental chair workbench, used a protractor to measure the angle between the laser tip and the horizontal plane at a 45° angle, ~ 0.5 mm away from the bottom plate of the bracket, fixed the hand, and moved the bracket horizontally for laser irradiation 60 s.
2. Flame treatment group (Group A2, A4, 4, IV): For this treatment, the base of the bracket was exposed to an external flame from an alcohol lamp (flame height approximately 4.5 cm, external flame averaging temperature 500 ℃) until turning red and remains 30 s, then it was cooled using cold distilled water. Adhesive removal from the bracket base was maximized with a probe.

### Bracket re-bonding

New brackets and reused brackets were rebonded to the teeth using a conventional bonding method in Group 2, 3, 4, II, III and IV.

### SBS and ARI detection

Bonding and re-bonding samples were transferred into a 37 °C water bath for 24 h (303–00 A, Shanghai Kuntian Experimental Instrument Co., Ltd, Shanghai, China), followed by exposure to 500 cycles of heating and cooling to 5 °C ± 2 °C and 55 °C ± 2 °C for 30 s at each temperature with the standard procedure of the machine (TC-501 F, Suzhou Weier Laboratory Supplies Co., Ltd, Suzhou, China) [20].

1. Shear bond strength (SBS) analysis was conducted by embedding the bonded bracket sample in a resin base platform attached to the test stand of a universal testing machine (3367, Instron, Boston, USA). The loading head of this universal testing machine was then positioned parallel to the base of the bracket and the cutting end of the bracket was loaded at a continuous 1 mm/min rate from the crown to the root until the detachment of the bracket. Maximum shear resistance for each group of isolated teeth was then recorded, with the SBS being calculated according to the premolar bracket base area as follows: SBS (Pa) = shear resistance (N)/bracket base area (m^2^).
2. ARI values for the enamel surfaces of individual teeth following bracket de-bonding were assessed using a stereo microscope (220,670, Olympus, Tokyo, Japan). The ARI scores were assigned as follows [21]: 0 points indicates the absence of any remnant adhesive on the tooth surface; 1 point indicates < 50% remnant adhesive on the tooth surface; 2 points indicates ≥ 50% remnant adhesive on the tissue surface; and 3 points represents all adhesive remaining on the tooth surface.

### SEM and EDS detection

1. Sample preparation: 4 metal brackets and 4 ceramic brackets were bonded to the 8 premolars in Groups A1, A2, A3 and A4 respectively. The brackets were removed with de-bonding pliers, and the residual adhesive was removed from the bracket bases using an Er: YAG laser or a flame in the same way.
2. Observation: For microscopic analysis of the bases in Group A, an additional 2 new metal brackets and 2 new ceramic brackets were prepared. These samples were affixed to the specimen bench and gold was applied with an ion spraying instrument (Jfc-1100, Shimadzu, Tokyo, Japan) (current: 5–10 mA, voltage: 1.2 kV, gold spraying time: 5 min). A scanning electron microscope (EVO 18, ZEISS, Oberkochen, Germany) with a vacuum launching field was then used to assess the morphology of both metal and ceramic bracket base. Residual components were detected on the ceramic bracket base via an energy-dispersive X-ray spectroscopy (EDS, Oxford, UK).

Based on the results of elemental content in the preliminary experimental, according to the method for calculating the sample size, it was calculated that the minimum required sample size for each is 16 by PASS 2021 software for sample size estimation

$$\left( n={\varPsi }^{2}\left[\sum _{i=1}^{k}{{s}_{i}}^{2}/k\right]/\left[\sum _{i=1}^{k}{\left(\stackrel{-}{{X}_{i}}-\stackrel{-}{X}\right)}^{2}/(k-1)\right] \right).$$

Anticipating a dropout rate of 20%, we therefore decided to include 20 sites in each group. For each ceramic bracket base, 10 sites were selected, with 2 brackets per group, thus totally 20 sites for EDS elemental content analysis.

### Statistical analysis

Data were analyzed with SPSS 24.0. Shear strength measurements were reported as means ± standard deviation. If the data were normally distributed with homogeneity of variance, they were compared with one-way ANOVAs with the Student–Newman–Keuls test for post hoc comparisons among groups. Otherwise, non-parametric tests were used. ARI scores were analyzed with the Kruskal-Wallis test. *P* < 0.05 served as the threshold of significance.

## Results

### SEM and EDS analysis of bracket bases

**Fig. 2:**
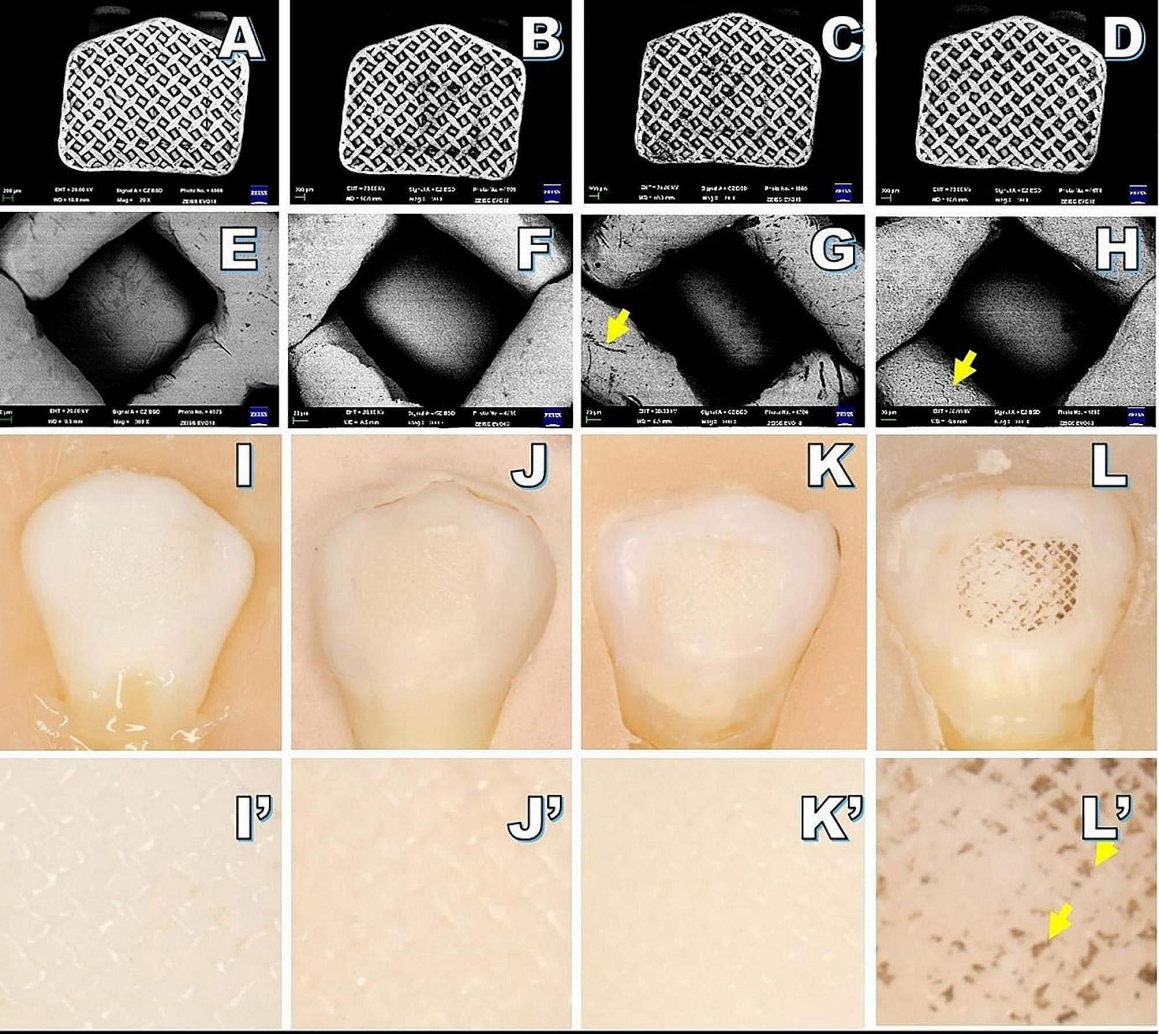
SEM images (×20/300 magnification) and images of the remnant adhesive on the enamel surface in the initial bonding and three re-bonding groups when using metal brackets. **A**, **B**, **E**, and **F** correspond to new bracket samples. **I** and **I**’ represent the initial bonding group. **J** and **J**’ represent the re-bonding group using a new bracket. In the Er: YAG laser group (**C**, **G**, **K**, and **K**’), fine nicks were observed, as indicated with arrows. In the flame group (**D**, **H**, **L**, and **L**’), samples exhibited evidence of abscission and black oxides, as indicated with arrows

**Fig. 3:**
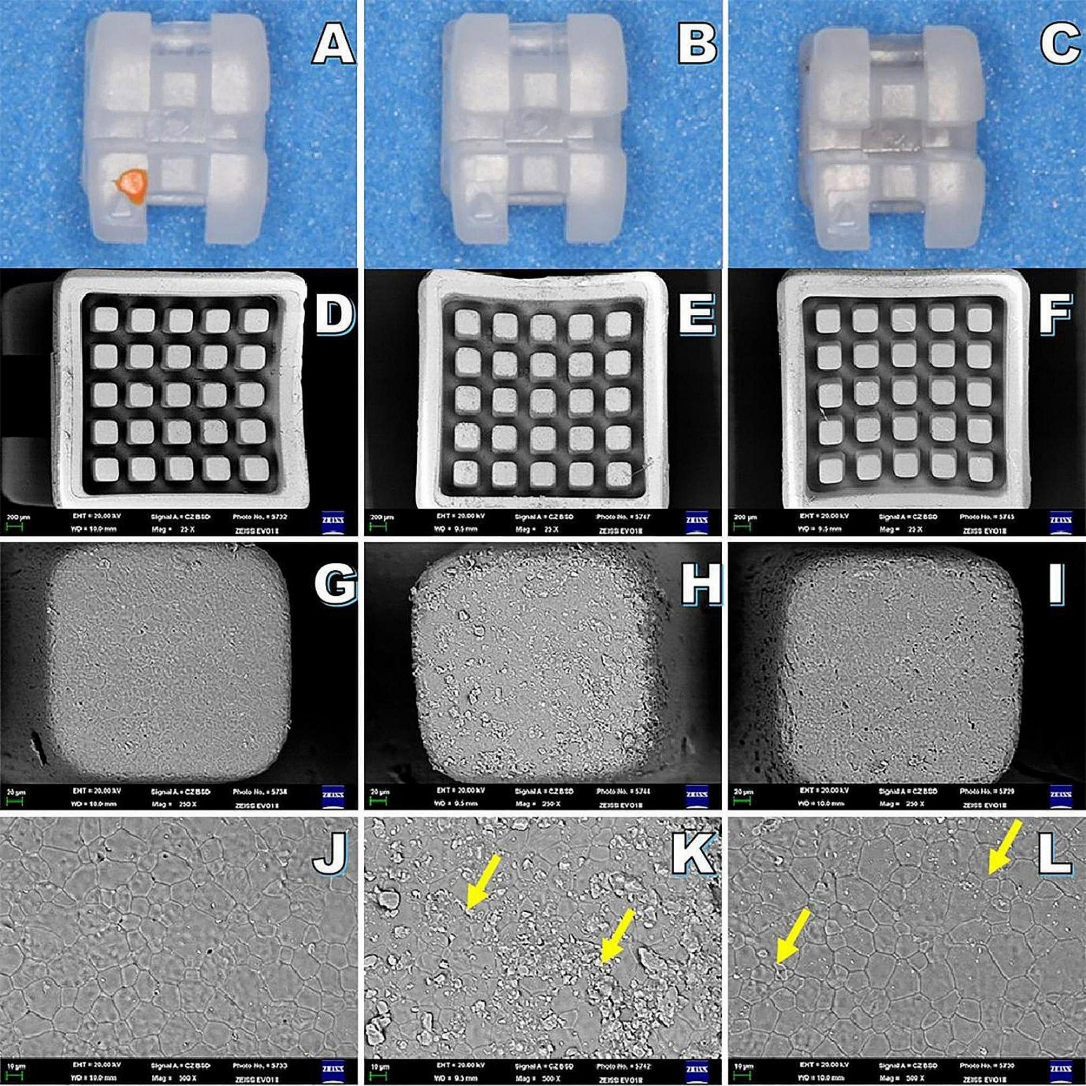
Ceramic brackets and SEM images (×25/250/500 magnification) for the study groups. **A**, **D**, **G**, and **J** correspond to the new bracket samples. **B**, **E**, **H**, and **K** correspond to the Er: YAG laser group, revealing adhesive as indicated with arrows. **C**, **F**, **I**, and **L** demonstrate that samples from the flame group exhibited lower levels of remnant adhesive, as noted with arrows

**Fig. 4:**
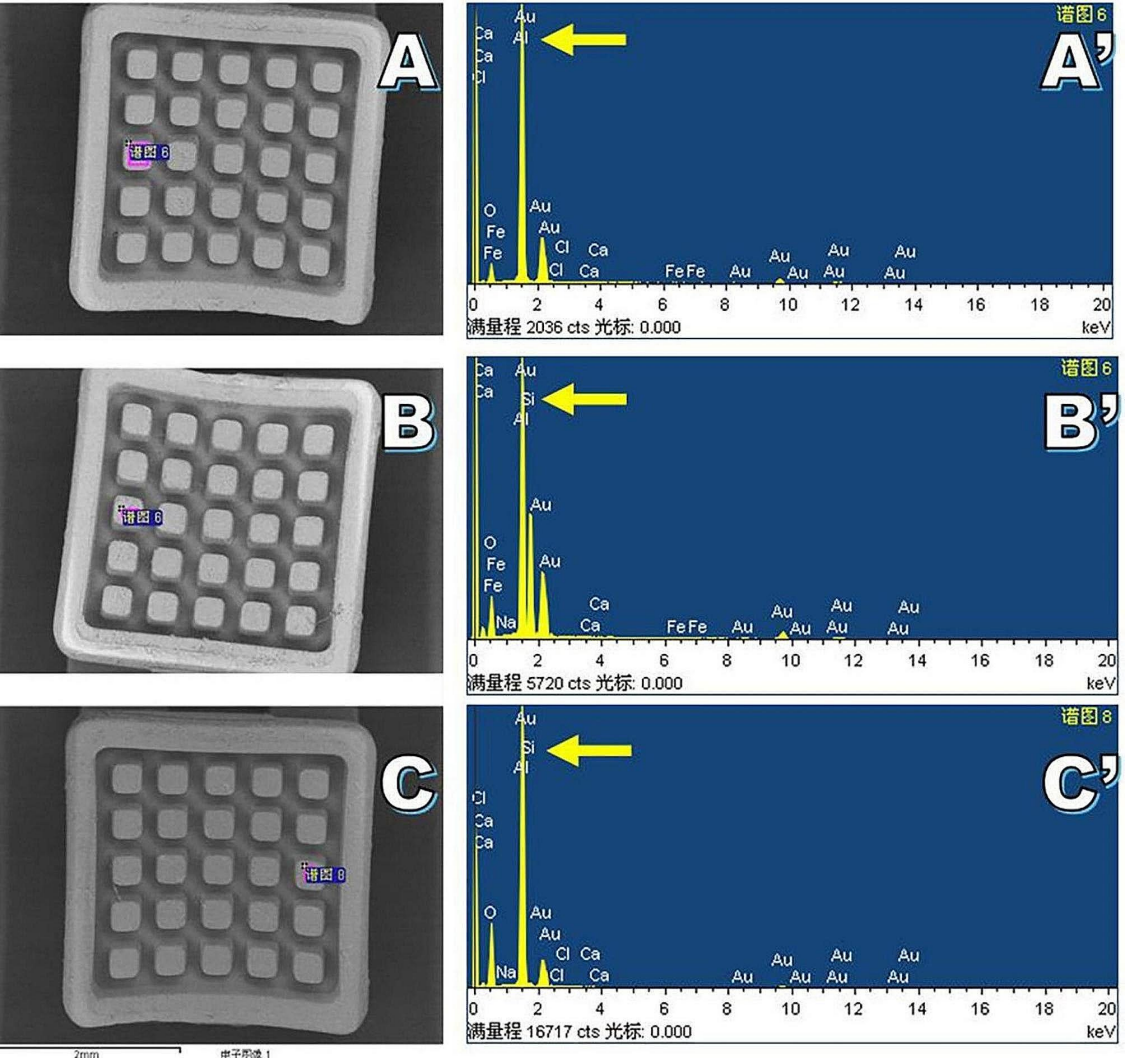
EDS images of the base of ceramic brackets from the treatment groups. **A**, **A**’: new bracket; **B**, **B**’: Er: YAG laser group; **C**, **C**’: flame group. The samples from the laser and flame groups contained silicon, as indicated with arrows

In the metal bracket, SEM analysis of the flame group revealed evidence of mesh abscission without detachment, while only fine nicks were detected in laser group (Fig. 2). In ceramic brackets, the bases of laser group exhibited greater amounts of adhesive residue compared to flame group (Fig. 3). A significantly higher level of silicon elemental content was detected in ceramic bracket bases from laser group (5.60 ± 2.73) relative to the flame group (0.90 ± 0.60) (*P* < 0.05, Table 1; Fig. 4). No conspicuous variations in color were observed in the new brackets or those treated with the laser. No damage was apparent in the laser group for bracket bases consisting of either metal or ceramic materials.

**Table 1:** EDS comparisons of the elemental content in ceramic bracket bases

Group		Element		F	*P*
	Al	O	Si		
New bracket	52.75 ± 0.13	47.02 ± 0.04	0.03 ± 0.37	34.27	< 0.001
Er: YAG	46.50 ± 3.06*	47.77 ± 0.37*	5.60 ± 2.73*	33.35	< 0.001
Flame	51.73 ± 0.70	47.12 ± 0.84	0.90 ± 0.60	34.45	< 0.001

Note: *A significant difference was detected between the Er: YAG laser group and the flame and new bracket groups

### Analysis of shear bond strength (SBS)

No significant differences in bonding strength were detected between Group 1 (24.95 ± 5.14 MPa) and Group 2 (23.61 ± 7.19 MPa) or between Group I (16.49 ± 7.11 MPa) and Group II (16.29 ± 8.34 MPa) (*P* > 0.05). However, a significantly lower bonding strength was observed in Group 4 (12.54 ± 6.55 MPa) (*P* < 0.05). When re-bonding was performed using recycled ceramic brackets, the SBS of the Group III (15.57 ± 8.47 MPa) was higher than that for the Group IV (14.71 ± 5.95 MPa), although the difference was not significant (*P* > 0.05, Tables 2 and 3).

**Table 2:** Shear bond strengths values for metal brackets

Group	N	SBS (MPa)	F	*P*
		Mean ± SD		
Initial bonding	20	24.95 ± 5.14	16.599	< 0.001
New bracket re-bonding	20	23.61 ± 7.19		
Er: YAG	20	26.13 ± 8.25		
Flame	20	12.54 ± 6.55*		

*P* < 0.05 was indicative of a significant distance

Note: *The flame group exhibited a significant difference relative to the laser and new bracket re-bonding groups

**Table 3:** Shear bond strengths values for ceramic brackets

Group	N	SBS (MPa)	F	*P*
		Mean ± SD		
Initial bonding	20	16.49 ± 7.11	0.25	0.86
New bracket re-bonding	20	16.29 ± 8.34		
Er: YAG	20	15.57 ± 8.47		
Flame	20	14.71 ± 5.95		

*P* > 0.05 indicates the absence of any significant difference

### Analysis of residual adhesive on the enamel surface and adhesive remnant index (ARI) values

**Fig. 5:**
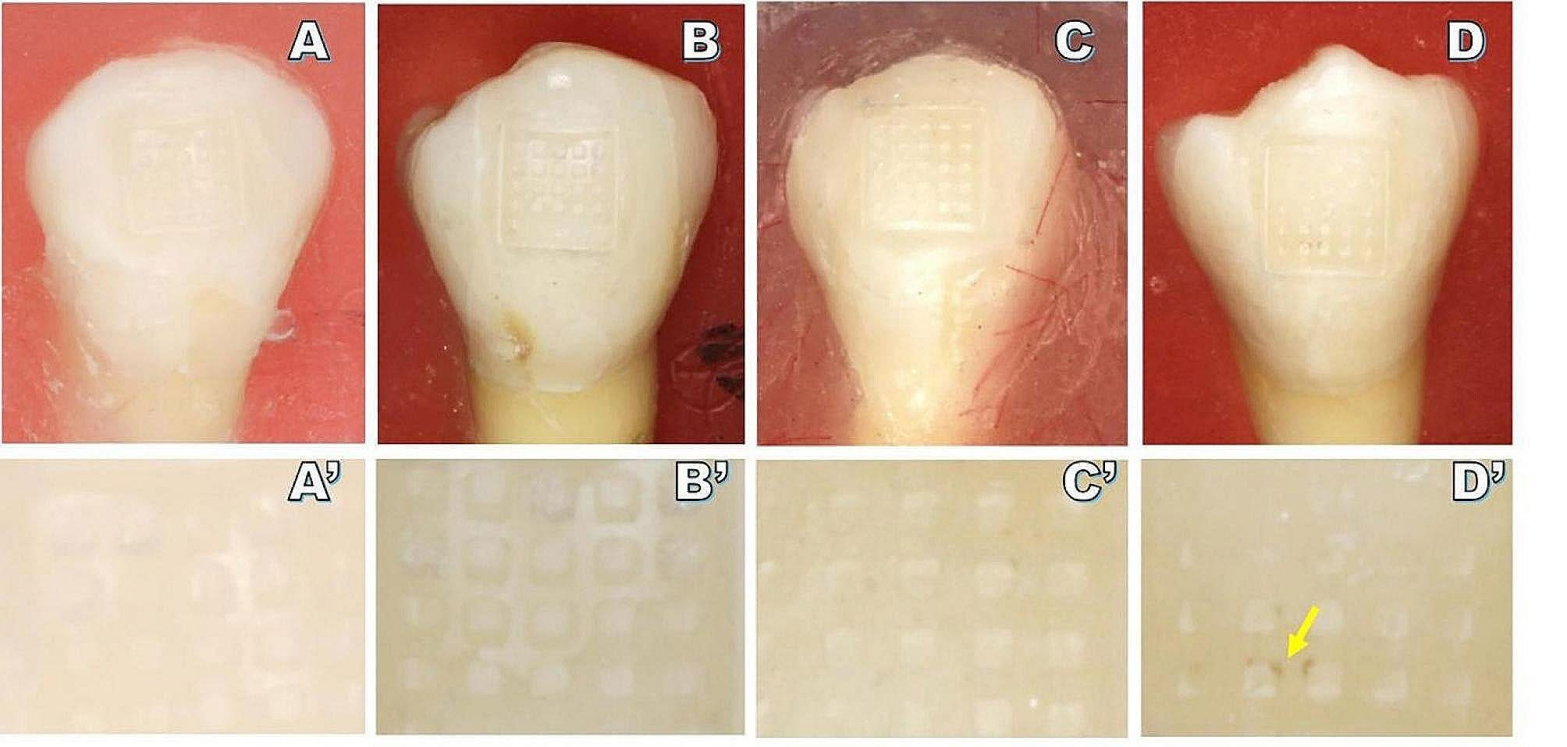
Images of remnant adhesive on the enamel surface for initial bonding and in the three re-bonding groups when using ceramic brackets. **A**, **A**’: initial bonding group; **B**, **B**’: new bracket re-bonding group; **C**, **C**’: Er: YAG laser group; **D**, **D**’: black oxides were evident in samples from the flame group, as indicated with the arrow

In Groups 1, 2, I and II, the surfaces of adhesive remnants on the enamel were clean and clear. In contrast, the residual adhesive surfaces in Group 4 exhibited black oxides (as indicated by the arrows in Figs. 2 and 5). When using recycled metal brackets, significant differences in ARI scores were observed among different groups (*P* < 0.05), with the ARI scores for the Group 4 (2–3 points) differing from those of the other three groups (Table 4). However, similar ARI scores were observed in all groups of recycled ceramic brackets (Table 5).

**Table 4:** ARI scores for metal brackets

Group	N	ARI				*χ* ^*2*^	*P*
		0	1	2	3		
Initial bonding	20	6	8	4	2	8.398	0.038
New bracket re-bonding	20	7	5	3	5		
Er: YAG	20	7	6	3	4		
Flame*	20	2	4	5	9		

*P*< 0.05 was indicative of a significant distance

Note: *The flame group exhibited a significant difference relative to the laser and new bracket re-bonding groups

**Table 5:** ARI scores for ceramic brackets

Group	N	ARI				*χ* ^*2*^	*P*
		0	1	2	3		
Initial bonding	20	0	6	7	7	0.390	0.999
New bracket re-bonding	20	0	5	7	8		
Er: YAG	20	0	5	7	8		
Flame	20	0	6	6	8		

*P* > 0.05 indicates the absence of any significant difference

## Discussion

The reuse of both metal and ceramic orthodontic brackets is common in orthodontic clinics. Utilizing a flame to dissolve residual adhesive material on the brackets can weaken the overall process of re-bonding and result in bracket discoloration [5]. Recently, dental lasers have been deployed with increasing frequency in orthodontic treatment including enamel etching [13], laser-assisted bracket removal [22], etc. Although laser has been used for the removal of residual adhesive from the tooth surface, the efficacy of the laser as a tool to remove residual adhesive from debonded metal or ceramic brackets is still not well understood. Therefore, compared with flame-based method, Er: YAG laser treatment strategy was applied to know how it affect bracket bonding strength relative to initial and re-bonding strength.

SEM revealed that the mesh surfaces of new metal brackets were smooth, whereas the mesh surfaces following Er: YAG laser treatment were narrow and rough without any residual adhesive. The impact may be due to the absorption of energy by water during laser treatment, resulting in local explosions, leading to the micro-roughening of the metal bracket base, thereby improving the overall bonding strength, which is similar to what has been reported by Chacko et al. [22] and Ishida et al. [17]. Ishida et al. removed the adhesive with an Er, Cr: YSGG laser and also found that this yielded a partially smooth mesh surface on the bracket base. In this study, black oxides were evident on the residual adhesive surfaces from the flame treatment group, consistent with the presence of some residual adhesive having been present and formed black oxides after burning. However, more black oxide formation was evident for the metal bracket group as compared to the ceramic bracket group, potentially consistent with the ability of heat to drive the formation of chrome-carbide compounds as visible evidence of metal stripping in SEM images. This may result in brackets that are more vulnerable to tarnishing and corrosion, increasing the probability of bracket detachment in the following orthodontic treatment [23]. More residual adhesive was present in the base of ceramic brackets treated by Er: YAG laser, without any significant change in the shape of the bracket. EDS analysis of the elemental content in the bases of the ceramic brackets revealed significantly more Si in the laser treatment group (5.60 ± 2.73) relative to the new bracket group (0.03 ± 0.37) and the flame group (0.90 ± 0.60). While water was sprayed during the laser treatment procedure, it is possible that it was not sufficient to completely remove the residual adhesive which absorbs the energy of the laser. The flame treatment method, in contrast, caused the combustion of adhesive, after which it was rinsed with water and treated with pressurized gas, leading to lower levels of residual adhesive relative to the laser group.

There have been some reports suggesting that rebonding is associated with improvements in bonding strength [24], while others have failed to detect the effect [25]. In the study, we compared SBS values of initial bonding to re-bonding utilizing both new and recycled metal or ceramic bracket. More detailed analysis of re-bonding strength for metal brackets revealed that there was a non-significant increase in shear strength in the Er: YAG laser treatment group (26.13 ± 8.25 MPa) relative to the initial bonding group. And no significant differences in SBS values were detected when comparing the three ceramic re-bonding groups, with the highest SBS being exhibited for new brackets (16.29 ± 8.34 MPa), followed by recycled brackets treated by Er: YAG laser (15.57 ± 8.47 MPa), and with flame (14.71 ± 5.95 MPa). A shear strength of 5.9–7.8 MPa has previously been reported to be sufficient for clinical bracket bonding [2]. The use of a laser (300 mJ, 20 Hz) to remove adhesive from the base of recycled brackets in the present study yielded higher levels of bonding strength as compared to past reports. Mirhashemi et al. [14] found that an Er: YAG laser (4 W, 10 Hz) was effective for the recycling of ceramic brackets in their study. In another study, the use of an Er, Cr: YSGG laser (3.75 W, 20 Hz) to treat the adhesive on the base of metal brackets resulted in a bonding strength of 10.7 ± 2.27 MPa [15]. Still others have described higher SBS values by laser (8.33 ± 2.51 MPa) than new brackets (250 mJ, 25 Hz) [26]. A similar report that ceramic bracket bases treated with an Er: YAG laser (5.5 W, 275 mJ, 20 Hz) for re-bonding led to relatively high bonding strength levels (13.4 ± 2.93 MPa) [27]. These inconsistent results may be due to the variability in terms of laser settings in the previous studies.

In the present analysis, metal brackets exhibited higher initial and re-bonding strength values relative to ceramic brackets, other than in the flame treatment group. Bonding strength may be more robust when bonding fractures occur in both the enamel-adhesive and the bracket-adhesive interface, rather than being confined to a particular interface. However, as excessively high bonding strength can complicate the process of bracket removal and result in damage to the enamel.

For metal brackets in the flame treatment group, residual adhesive remained present on the surface of the tooth, while black oxide was found on the base of the bracket. The ARI values mainly ranged from 2 to 3, suggesting that more than 50% of the tooth surface was covered by residual adhesive and that failure occurred at the bracket-adhesive interface. Some portion of the base of the bracket may have flaked off and adhered to the residual adhesive surface following flame exposure. However, widely distributed adhesive residues were evident in both the Er: YAG laser and new bracket treatment groups, suggesting the occurrence of failure at the enamel-adhesive interface and the bracket-adhesive interface without any significant difference between these groups. For ceramic brackets, ARI scores did not differ significantly among different groups. Yassaei et al. [27] also reported a mixed failure mode, with fracture occurring at both interfaces. In contrast, Ishida et al. [17] just observed the bonding fracture interface for the enamel-adhesive interface following Er, Cr: YSGG base re-treatment. These discrepant findings may be related to variable laser types and laser treatment parameters. Studies of the effects of other Er: YAG laser settings on re-bonding following bracket therapy are thus needed.

## Conclusions

In summary, the results of this study revealed that Er: YAG laser treatment (300 mJ, 20 Hz) was superior to flame treatment as a means of removing residual adhesive from bracket surfaces without any staining of metal or ceramic brackets. SBS values and ARI scores for new brackets were comparable to those of the brackets recycled using Er: YAG laser treatment. Therefore, the Er: YAG laser treatment strategy represents an attractive approach to recycling debonded orthodontic brackets.

### Electronic supplementary material

Below is the link to the electronic supplementary material.

Supplementary Material 1

## Data Availability

Data is provided within the manuscript or supplementary information files.
